# Recanalisation of an axillary vein occlusion jailed by a migrated cephalic arch stent-graft using the TruePath chronic total occlusion drilling device

**DOI:** 10.1186/s42155-020-0098-5

**Published:** 2020-01-14

**Authors:** Ankur Patel, Shaun Xavier Ju Min Chan, Kun Da Zhuang

**Affiliations:** Department of Vascular & Interventional Radiology, Singapore General Hospital, Outram Road, Singapore, 169608 Singapore

**Keywords:** Dialysis access, Recanalisation, Stent graft, TruePath

## Abstract

**Background:**

Stent placement in the cephalic arch is being used with increasing frequency. Late complications of bare metal and stent grafts in dialysis access, in particular stent migration, are often under-reported and can lead to compromise of future dialysis circuits.

**Case presentation:**

A 52-year-old man developed acute arm swelling 2 days after creation of a left arm brachio-basilic arteriovenous graft. The axillary vein was found to be jailed by a previously deployed cephalic arch stent graft which had migrated into the subclavian vein. There was failure to cross through the fabric of the stent graft using conventional chronic total occlusion wires and techniques. A TruePath device was used successfully to cross through the fabric of migrated cephalic arch stent graft and recanalise the short subclavian-axillary vein occlusion.

**Conclusion:**

The adapted use of a drilling chronic total occlusion device to drill through the fabric of migrated stent graft was performed successfully to allow complete recanalisation of the occluded axillary vein.

## Introduction

Stent-graft (SG) placement for cephalic arch stenosis in dysfunctional dialysis access is an option for patients, and a recent systematic review suggests that it may be more durable in the short term compared to angioplasty alone (Miller et al. 2018; D’Cruz et al. 2019). Precise placement of SGs in the cephalic arch, particularly the terminal segment near or at the junction with the axillary vein, can be difficult. Due to the angle at which the cephalic arch joins the axillary vein, over or under stenting of the stenosis is not uncommon. Central migration of SGs may result in subclavian and axillary vein occlusions, compromising future dialysis access options in the ipsilateral arm and may cause symptoms of central venous occlusion. We report the novel use of a diamond tipped chronic total occlusion (CTO) drilling device, TruePath (Boston Scientific, MA, USA), to recanalise an occluded axillary-subclavian vein segment that had been jailed by a migrated cephalic arch SG.

## Case report

**Fig. 1:**
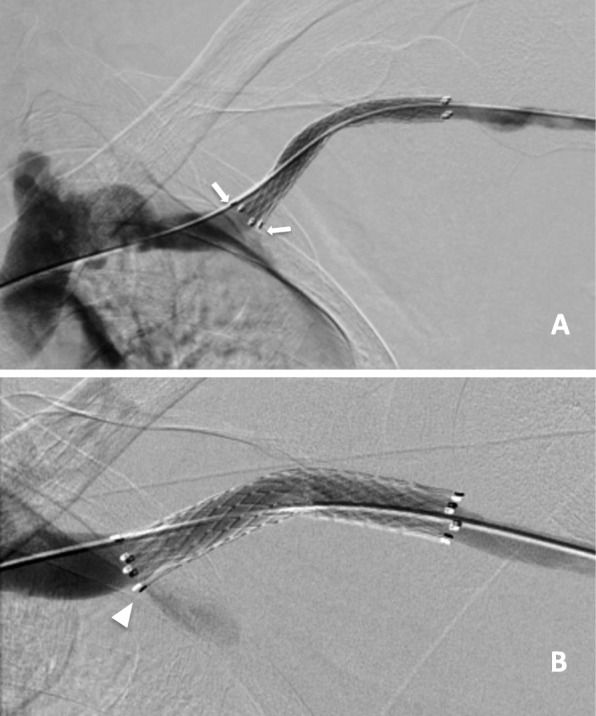
Cephalic arch venograms. **a** Immediately after deployment of the 8 × 60 mm Fluency stent graft showing precise positioning (arrows) of the central end of stent flush with the superior wall of the subclavian vein. **b** 5 months after stent deployment, the inferior edge of the central end of the stent is back-walling the inferior wall of the subclavian vein (arrowhead) resulting in jailing of the axillary vein

A 52-year-old man was on long term haemodialysis following a failed renal transplant. Non-maturing left radiocephalic and brachiocephalic arteriovenous fistula (AVF) initially created were abandoned. He underwent hemodialysis via a right brachiocephalic arteriovenous graft (AVG) but required multiple interventions to maintain patency. During a thrombolysis procedure, venoplasty of a cephalic arch occlusion resulted in a focal rupture for which conservative management with balloon tamponade was unsuccessful. An 8 × 60 mm Fluency SG (Bard, NJ, USA) was deployed in the cephalic arch to manage the rupture. Following deployment, the SG showed no protrusion into the subclavian vein (Fig. 1). On a subsequent venoplasty procedure 5 months later, the central end of the stent had migrated into the subclavian vein resulting in jailing of the axillary vein with consequent occlusion of that segment (Fig. 1). The patient remained asymptomatic. After further interventions over a two-and-a-half year period, this AVG was eventually abandoned.

**Fig. 2:**
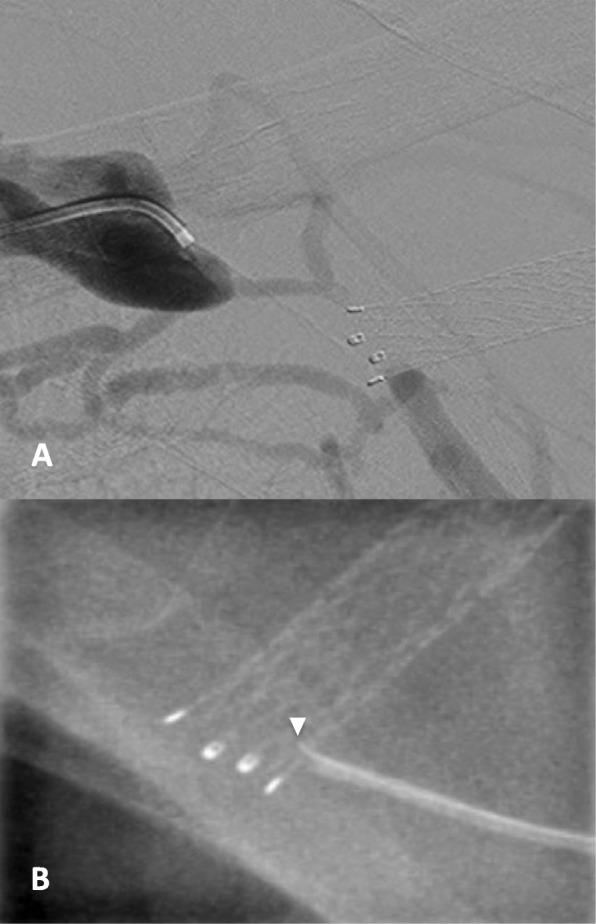
**a** Venogram showing short segment occlusion of the subclavian vein from the protruding cephalic arch stent graft. **b** Attempted antegrade wire crossing with the Winn 200 T wire (arrowhead), which was unable to penetrate through the stent graft fabric via the antegrade approach

A new left loop brachiobasilic AVG was created inadvertently and the patient developed left arm swelling 2 days post-surgery. A graftogram showed complete occlusion of the axillary-subclavian vein from the migrated cephalic arch SG. Attempts to cross the axillary vein occlusion using 0.035″ standard guide wires, 0.018″ glide wires (Terumo, Tokyo, Japan), a 0.014″ Winn 200 T (Abbott Vascular, CA, USA) and 0.018″ Victory 25 g (Boston Scientific, MA, USA) CTO wires were all unsuccessful due to the inability to traverse the fabric of the Fluency SG (Fig. 2). Due to the steep angulation, sharp recanalization was not attempted in view of potential injury to the subclavian artery.

In view of the limited access options, a further attempt was made using the TruePath CTO drilling device. Under ultrasound guidance, 6F and 4F sheaths were inserted into the left common femoral vein and the venous limb of the left AVG respectively. Venography revealed a 2 cm subclavian vein occlusion adjacent to the central end of the migrated SG (Fig. 2). Attempted retrograde crossing of the occluded segment was unsuccessful and complicated by self-limiting guidewire perforations.

**Fig. 3:**
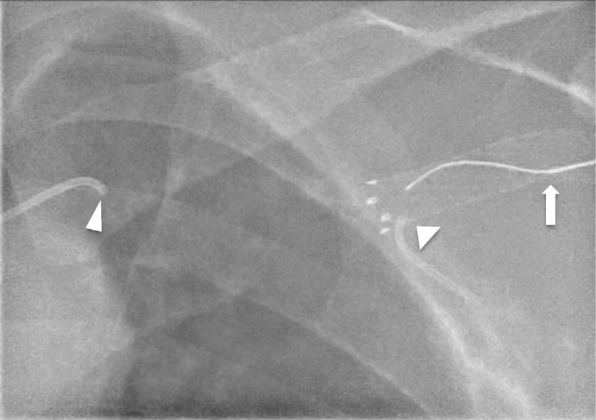
Fluoroscopic image of the occluded left cephalic stent graft, which was accessed with a 21G micropuncture needle under ultrasound guidance. The tip of the needle (arrow) within the occluded stent lumen, with a V18 wire directed centrally. Tips of the antegrade axillary vein (broad arrowhead) and retrograde subclavian vein (narrow arrowhead) 4F Berenstein catheters as shown

**Fig. 4:**
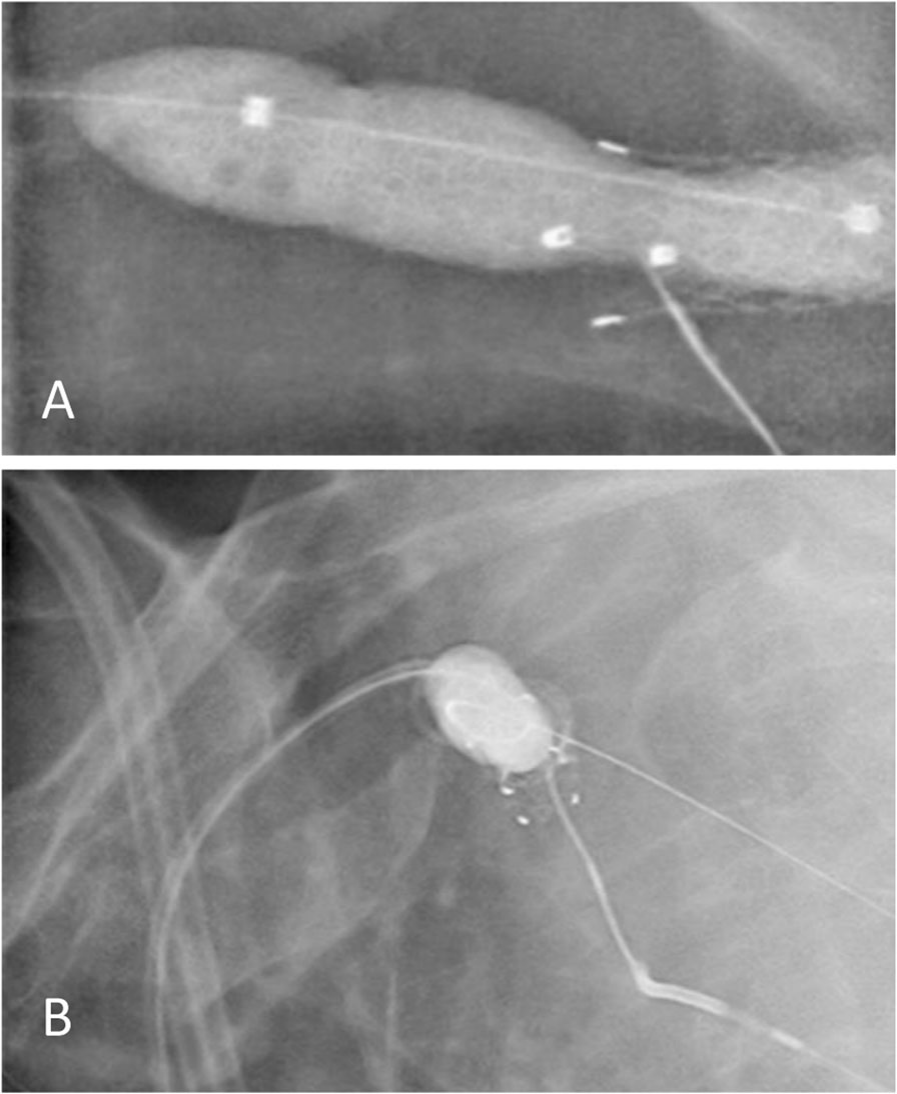
Fluoroscopic images showing the drilling tip of the TruePath device (supported by a 4F Berenstein catheter and 0.018″ CXI support catheter),penetrating through the fabric of the cephalic arch stent graft using the in-situ 10 × 40 mm Advance LP balloon as a target. **a** Conventional anterior-posterior (AP) frontal projection, and **b**, a near lateral projection with steep cranio-caudal angulation to create a ‘tunnel’ view to confirm precise directionality of the TruePath drilling tip

**Fig. 5:**
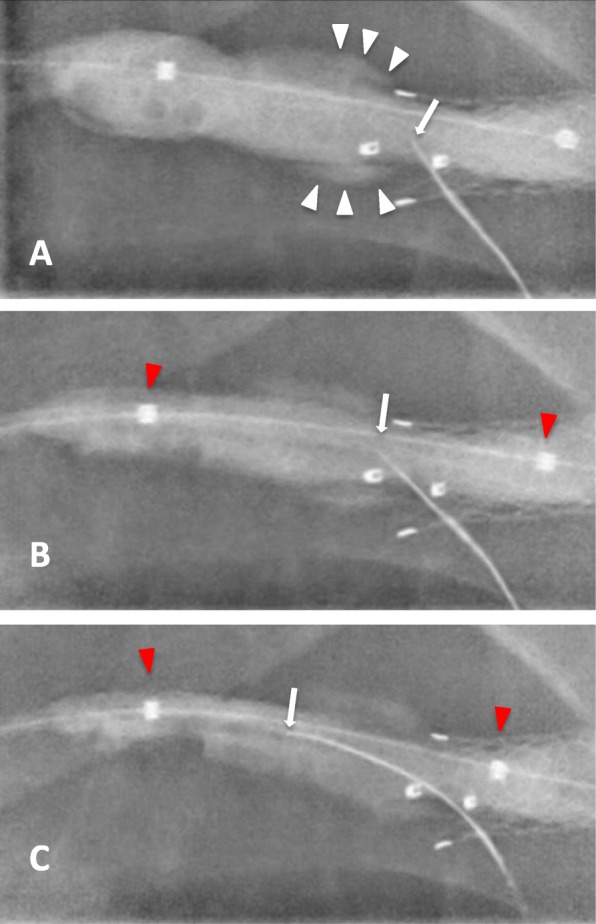
Sequential fluoroscopic images showing the drilling tip (arrows) of the TruePath device as it punctures the inflated angioplasty balloon within the cephalic arch stent graft with, **a** rapid deflation of the balloon with contrast spill (white arrowheads) confirming successful entry into the stent lumen. **b** and **c** Progressive advancement of the TruePath wire deeper into the balloon with tandem withdrawal of the balloon (red arrowheads) centrally together with the wire tip

**Fig. 6:**
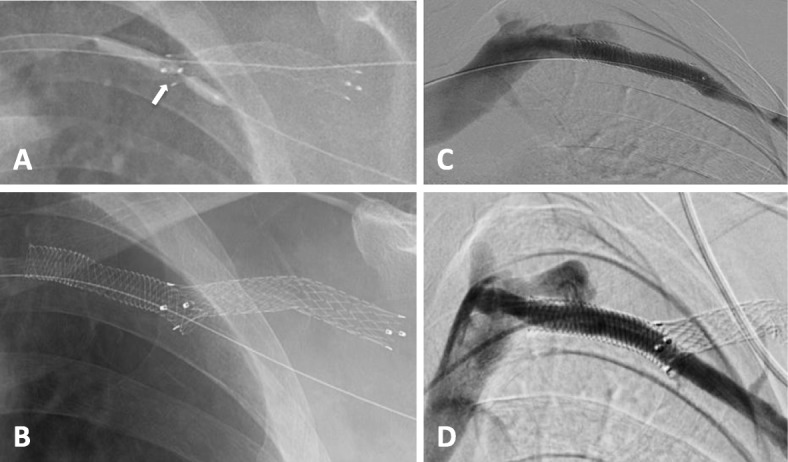
**a** Fluoroscopic image showing focal ‘wasting’ of the pre-dilatation angioplasty balloon (arrow) through the stent wall. **b** Post Supera stent deployment showing satisfactory stent opening through the cephalic arch stent graft. **c** Antegrade venogram showing good flow through the Supera stent into the subclavian vein with no residual stenosis. **d** Retrograde venogram performed 6 months after stent deployment showing a widely patent Supera stent and axillary vein

Via a 21G micropuncture needle (Cook Medical, IN, USA), the peripheral end of the occluded SG was accessed (Fig. 3). Using a V18 Control wire (Boston Scientific), luminal traversal of the occluded SG and subclavian vein segment was achieved. The V18 wire was snared via the left common femoral sheath using a looped 0.025″ Glidewire (Terumo, Tokyo, Japan) to achieve through-and-through access. Thereafter, a 10 × 40 mm Advance LP (Cook Medical, IN, USA) angioplasty balloon was inflated within the recanalised SG to serve as a subsequent target. Via the AVG 4F sheath, using co-axial support of a 4F Berenstein catheter (Cordis, CA, USA) and a 0.018″ CXI support microcatheter (Cook Medical, IN, USA), the TruePath CTO drilling device was advanced along the basilic and axillary vein up to the point of occlusion. Under orthogonal fluoroscopic guidance (Fig. 4), the SG ePTFE fabric was penetrated with the TruePath drilling tip towards the inflated Advance LP balloon. Rapid deflation of the punctured balloon indicated successful SG entry (Fig. 5). The TruePath wire tip, now within the ruptured balloon, was advanced centrally with the balloon in tandem. Thereafter, the CXI support catheter was tracked over the TruePath wire and exchanged for a 300 cm V18 Control wire. The balloon was then removed via the left CFV sheath and the V18 Control wire was snared out. Via the femoral sheath, the 5 × 40 mm Sterling balloon was used to pre-dilate the axillary-subclavian occlusion as well as the SG fabric (Fig. 6). Also, via the femoral sheath, vessel preparation was performed with a 7 × 40 mm Mustang balloon (Boston Scientific) followed by a 7 × 40 mm Supera stent (Abbott Vascular, CA, USA) which was deployed across the recanalised subclavian segment, through the SG and into the axillary vein. Completion angiography showed no residual stenosis or recoil of the newly stented segment (Fig. 6).

Left arm swelling improved significantly over the 3 days. Unfortunately, late infection of the AVG developed and was explanted 28 days post intervention. New long-term AVG dialysis access was created in the left lower limb. Six months after left subclavian-axillary vein stenting, an opportune catheter angiogram via his left groin AVG demonstrated continued patency, and without recoil, of the stented segment (Fig. 6).

## Discussion

Cephalic arch stenosis affects up to 34% of dysfunctional brachiocephalic AVFs (Bennett et al. 2015). The use of stents, in particular SGs, to treat recurrent cephalic arch stenosis has been increasing due to low primary patency rates of balloon angioplasty and an increasing body of evidence demonstrating superior patency rates of stents (Miller et al. 2018; D’Cruz et al. 2019). Despite appropriate sizing and accurate deployment of the Fluency SG, central stent migration may result in jailing of the axillary vein. Relative mobility of the cephalic arch segment and external compressional forces from the claviculopectoral and deltopectoral fasciae may contribute to SG migration. The problem of stent migration in the cephalic arch is probably under-reported as these patients tend to be asymptomatic (Sequeira 2016).

Conventional techniques of crossing the occlusion were unsuccessful due to the SG fabric preventing wire passage. Due to vascular anatomy and potential inadvertent subclavian artery injury, sharp recanalisation employing a straight needle was deemed high risk. Bench-testing using a demonstration unit of the TruePath device and Fluency SG demonstrated controlled and easy traversal of the drilling tip of the device through the SG fabric. TruePath is guidewire-mounted mechanical recanalization device with a rotating distal diamond tip designed for true-lumen crossing of peripheral arterial occlusions. We adapted the previously reported balloon puncture technique, for placement of dialysis access catheters in occluded internal jugular veins (Too et al. 2016) for this case. Once recanalised, stenting was mandated in view of the aetiology of this occlusion. A Supera stent was chosen for its crush-resistance properties, superior flexibility and kink resistance.

## Conclusion

The case report demonstrates the successful novel use of the TruePath device to drill through the fabric of a migrated cephalic arch stent graft which had jailed the axillary vein outflow causing acute arm swelling due to compromise of the axillary vein outflow. Migration of cephalic arch stent grafts are under-reported and can compromise future dialysis access options. The use of the TruePath device facilitated recanalization of the occluded axillary vein. The device and techniques used in this case can be utilised in similar situations, where conventional techniques would pose high risk of arterial injury or likely be futile.

## Data Availability

Further data and images regarding this clinical case are available upon request.

## Abbreviations

AVF: Arteriovenous Fistula
AVG: Arteriovenous Graft
CTO: Chronic Total Occlusion
SG: Stent Graft
